# Artificial Neural Networks and the Actiotope Model of Giftedness—Clever Solutions from Complex Environments

**DOI:** 10.3390/jintelligence11070128

**Published:** 2023-06-25

**Authors:** Shane N. Phillipson, Cindy Di Han, Vincent C. S. Lee

**Affiliations:** 1 Department of Education, Swinburne University of Technology, John Street, Hawthorn, VIC 3122, Australia; 2 Faculty of Education, Monash University, Wellington Rd, Clayton, VIC 3052, Australia; cindy.han@monash.edu; 3 Department of Data Science and Artificial Intelligence, Monash University, Wellington Rd, Clayton, VIC 3052, Australia; vincent.cs.lee@monash.edu

**Keywords:** Actiotope Model of Giftedness, artificial neural networks, complexity, research

## Abstract

Since its inception, the Actiotope Model of Giftedness (AMG) has provided researchers with a useful model to explain the development of exceptionality. Rather than a focus on the individual, the model postulates that exceptionality is the outcome of a system that includes complex interactions between an individual’s current level of talent and their internal and external environment. To date, however, the statistical techniques that have been used to investigate the model, including linear regression and structural equation modeling, are unable to fully operationalize the systemic nature of these interactions. In order to fully realize the predictive potential and application of the AMG, we outline the use of artificial neural networks (ANNs) to model the complex interactions and suggest that such networks can provide additional insights into the development of exceptionality. In addition to supporting continued research into the AMG, the use of ANNs has the potential to provide educators with evidence-based strategies to support student learning at both an individual and whole-school level.

## 1. Introduction

As Matthews and Jolly (2022) noted, the development of both special and gifted education developed alongside research into the nature, origins, and measurement of intelligence. Both specialized fields of education continue to use this research in order to maximize student academic outcomes. Despite the problematic nature of the term ‘gifted’ (Matthews and Jolly 2022), however, we now recognize that academic outcomes, including those judged to be exceptional, depend on both cognitive and non-cognitive factors such as good teaching, effective learning strategies, and personality. Today, teachers in the U.S. are able to draw on several models to describe the development of exceptionality, including the Piirto Pyramid of Talent Development (Piirto 2021) and the Schoolwide Enrichment Model (Renzulli and Reis 2021).

Two lesser-known models of talent development are yet to impact education, including Françoys Gagné’s (2013, 2018, 2021) Differentiated Model of Giftedness and Talent (DMGT) and Albert Ziegler’s (Phillipson et al. 2013; Ziegler 2005) Actiotope Model of Giftedness (AMG). Although a comprehensive review of Gagné’s DMGT is beyond the scope of this article, the model is yet to be externally validated or fully implemented as a model of gifted education (Gagné 2021; Phillipson and Ziegler 2021). As Phillipson and Ziegler argued, the DMGT can be criticized for its reliance on the identification of a number of innate ‘gifts’ and the elements of ‘chance’ in the development of exceptionality.

Since its initial explication (Ziegler 2005), the AMG has inspired a number of research efforts to operationalize the model and to explore its generalizability across different educational and cultural contexts. At its heart, the AMG theorizes that exceptionality is the outcome of a complex system that includes the person interacting with their material, social, and informational environment (Ziegler et al. 2013). Rather than maintain a semblance of stability, the system needs to undergo periodic change in order to facilitate a trajectory toward exceptionality (Ziegler et al. 2013, 2017; Ziegler and Stoeger 2017). As Ziegler et al. (2013, p. 3) explained, the fundamental theoretical unit is an actiotope, defined as the material, social, and informational environment in which an individual actively interacts.·

Although Ziegler intended the model to explain the antecedents of exceptionality, it is not surprising that much of the research has focused on its usefulness to explain the academic achievement of students in primary and/or secondary education. The methods used in this research include statistical techniques such as linear regression and structural equation modeling (SEM), and while they have provided some insights into the actiotope, such techniques are not consistent with the theoretical basis of the AMG. We contend that the potential of the AMG to explain academic achievement (and, ultimately, exceptional performance) is hindered by such techniques and that new analytic methods are required.

In accordance with the objectives of this special issue, the purpose of this article is to examine further the interactions of environmental influences on the development of exceptionality. Specifically, we build on the theoretical basis of the AMG and argue that artificial neural networks (ANNs) provide an opportunity to better explore the complex and non-linear relationships between the components of the AMG and allow predictions to be made about the possible consequences of changes to an individual’s actiotope. Accordingly, we begin by briefly reviewing the theoretical basis of the AMG to describe the development of skills and knowledge within any domain. Keeping in mind that the AMG is explained fully elsewhere, we focus on the systemic features of the model. We then outline the research to date and show that despite efforts, much of this research has been unable to take into account the complexity within a learner’s actiotope.

Although a full description of ANNs are beyond the scope of this article, we provide a brief example of how ANNs have been used in other domains to model complexity and its capacity to make predictions. We conclude with a research framework that integrates this technology with the theoretical basis of the AMG.

In this article, we use the term *talent* to denote the continuous outcome of the actiotope related to the skills and knowledge required for a given domain. Thus, talent describes the outcome of a system in any given domain that can be measured at various times throughout the developmental process. In contrast, the terms *exceptionality* and *excellence* refer to the societal and/or peer evaluations of the quality of this talent. Despite its inclusion in the model’s full name, we avoid the use of the term ‘gifted’ because of its negative connotations (Matthews and Jolly 2022).

Of course, talent is likely to vary across time for any one individual. At the same point in time, talent will also vary from one individual to another. Given the limitations of measurement, the concept talent is best considered a latent variable and hence is imperfectly measured by indicators such as standardized test scores and/or other achievements in the domain.

## 2. Systemic Nature of the Actiotope Model of Giftedness

In order to clarify the integration of the AMG with ANNs, we begin with a vignette of a hypothetical student we have named Susie. As Ziegler has often stated (e.g., Ziegler et al. 2013), the ultimate objective of actiotope research would be the means to predict likely changes in Susie’s development, for example, because of changes in her environment.

*Susie is a Year 8 school student. Two years ago, Susie had ambitions to become an engineer. Her reading level was several years ahead of her classmates and she enjoyed learning mathematics. Today, Susie is now struggling academically and her parents and teachers are concerned that her mathematics achievement scores do not appear to match her potential. Furthermore, Susie’s parents and teachers agree that without some specific intervention, there is a danger that she may fall behind and not realize her dreams. The problem is: what intervention is needed? What is the likelihood that this intervention will raise her current mathematics score from 50% to 75%?*

In understanding Susie’s situation, parents and teachers are able to draw on the AMG (Phillipson et al. 2013; Ziegler 2005). As the nomenclature suggests, the AMG was first described in the context of ‘giftedness’ and gifted education. As we have already stated, Ziegler (2005) considered *exceptionality* the end-point of a long developmental process punctuated over time by progressive changes in the interactions between the learner and their environment (e.g., Ziegler and Phillipson 2012; Ziegler and Baker 2013; Ziegler and Stoeger 2017). In broad terms, changes in the environment are needed to facilitate growth in talent. Conversely, growth in talent will itself require changes in the environment. Ziegler and Baker (2013) categorized an individual’s immediate environment as neutral, positive, or negative, depending on its impact on the development of their talent.

The four main components of an individual’s actiotope include their action repertoire, goal states, exogenous and endogenous environment, and subjective action space (Ziegler et al. 2013). In the AMG, resources refer to the ‘means that can be used to obtain goals’ (Ziegler and Baker 2013, p. 26). Drawing on a broad research base, Ziegler and Baker preferred the term capital^1^ to refer to each of the 10 distinct elements within the educational and learning environments. Furthermore, actiotopes draw on these capitals in order to maintain *homeostasis* and to support *allostasis*.

In Susie’s context, action repertoire refers to the sum total of the action possibilities to which she has access, including her current levels of skills and knowledge in mathematics, for example. Susie’s goal states refer to her many (and possibly competing) goals, including her goal to be an engineer, wanting to please her parents, and the need to be accepted by her friends. Susie’s exogeneous environment refers to those capitals that *may* be used to support her learning, whereas endogenous environment refers to those capitals that are only used to support learning. Finally, Susie’s subjective action space refers to those actions that she uses to achieve her immediate objectives. For example, Susie chooses not to complete her school assignment because she fears being ostracized by her friends.

In adopting a systemic approach to the development of exceptionality, the interactions between the components of the system at any given time are in one of two states of regulation, namely *homeostatic* regulation and *allostatic* regulation (Ziegler and Baker 2013; Ziegler et al. 2017; Ziegler and Phillipson 2012; Ziegler and Stoeger 2017). During *homeostatic* regulation, the system is at a steady state and the developmental process essentially stalls. In other words, *homeostatic* actiotopes promote little or no growth in talent development.

In contrast, *allostatic* regulations move from one steady state to another adjusted steady state in response to challenges and stimuli. Thus, allostatic regulations are crucial for talent development. According to Ziegler and Baker (2013), ‘new resources must be activated in order to obtain [the next] stable state’ (p. 25). For example, Susie responds positively to her teacher’s challenge to regain her love of reading and challenge of competing in the school’s mathematics competition by making more effort in class and seeking out new friends. As her expertise develops, however, her current teacher may not be adequate to facilitate growth in talent, and a new teacher is needed to disrupt the homeostasis.

To summarize: during periods of *homeostasis*, when there are no changes to the learner’s environment and how they interact with the environment, growth in talent proceeds at a constant rate. These *homeostatic* periods are punctuated with rapid growth because of changes within Susie and/or her external environment. In contrast, periods of rapid growth that occur when the system is undergoing change are referred to as *allostasis*. According to Ziegler and Baker (2013), capitals are regulated in ways that support *homeostasis* (i.e., homeostatic regulation) or *allostasis* (allostatic regulation). Irrespective of the type of regulation, the system responds to changes in the availability of capitals.

In theorizing how these capitals affect each other and the outcomes of the system, Ziegler and Stoeger (2017) suggested that all of the capitals interact with each other in loops in which changes in any one of the capitals can initiate change in other capitals and potentially the outcome of the actiotope. In accordance with systems in general, the interactions can be of five types, including additive, multiplicative, positive feedback loops, negative feedback loops, and co-evolutions.

Returning to Susie, small but positive changes in her teacher’s demeanor may result in huge changes in Susie’s goals, her circle of friends, and her self-esteem. Furthermore, Susie’s parents may suggest she be tutored by someone recommended to the family. As Ziegler and Stoeger (2017) pointed out, the AMG suggests that it does not matter where the change occurs—just that *homeostasis* needs to be challenged if growth in talent can occur. In Susie’s situation, the new tutor may be all that is necessary to effect the change.

At the time, Ziegler and Stoeger (2017) cautioned that the systemic perspective of talent development is not amenable to direct empirical testing. Rather, research is limited to contexts in which capitals are not well interconnected. As such, however, a reductionist approach cannot fully reflect the finer points of a system that includes the full complement of interaction possibilities. As should be obvious at this stage of the article, we disagree with Ziegler and Stoeger’s initial assessment and suggest that ANNs offer a way to empirically test the AMG. As already outlined, we will briefly review the research evidence to support the theoretical basis of the AMG before describing ANNs and our framework to integrate the AMG with ANNs in order to overcome these limitations.

## 3. Research Based on the Actiotope Model of Giftedness

To date, the research inspired by the AMG can be categorized into three broad groups. The first group includes validation studies that focus on the generalizability of the ‘capitals’ across different contexts and measure the components of the actiotope. The second group examines the way that these components interact with each other within a system in order to promote the development of talent, particularly the academic achievement of students. A thorough examination of the validation and interaction studies will assist in establishing the usefulness of ANNs to study the AMG.

The third group of research studies assumes the utility of the AMG to describe the overall educational environment and to suggest possible interventions. They include ‘mapping’ the educational environments of families of highly achieving students (Vialle 2017) and identifying the impact of COVID-19 on the learning experiences of Australian parents and their children (Phillipson et al. n.d.). Other ‘mapping’ research includes the Australian gifted education environment (Phillipson and Ziegler 2021), Egypt (Ayoub et al. 2022), Oman (Hemdan et al. 2022), Saudi Arabia (Alamer and Phillipson 2020; Alfaiz et al. 2022), and Sudan (Bakhiet and Mohamed 2022), the practice of Russian universities toward gifted students (Skorobogatova and Melikhova 2021), the support practices of Australian parents of their young children (Richards et al. 2019), and the educational aspirations of Singaporean parents (Phillipson et al. 2019).

In terms of interventions, the AMG provides a framework for mentoring programs (Ziegler et al. 2021), the remediation of student underachievement (Gilar-Corbi et al. 2019a, 2019b), and teacher training programs (Kollmayer et al. 2020). Finally, the AMG has inspired re-conceptualizations of the interactions between environmental influences and personal characteristics of gifted students (Mudrak et al. 2020; Subotnik et al. 2019) and has provided the basis for including ‘embodiment’ in gifted education (Awad et al. 2020). A thorough review of research in this third group is beyond the scope of this article.

### 3.1. Validation Studies

Despite the systemic nature of the AMG, attempts to operationalize the model have tended to focus on measuring an individual’s educational and learning capitals. According to Ziegler and Baker (2013), the five exogenous (*economic*, *cultural*, *social*, *infrastructural*, and *didactic*) capitals may be used by the individual to support the development of talent. In contrast, the five endogenous (*organismic*, *actional*, *telic*, *episodic*, and *attentional*) capitals are exclusively used by the individual to support talent development. To the original five exogenous capitals, Phillipson et al. (2017) added a sixth they termed *aspirations* (Table 1).

Research to test the validation of the AMG began with studies involving German, Chinese, and Turkish secondary students (Vladut et al. 2013) and later involved post-secondary German students (Vladut et al. 2015). In broad terms, participants reliably evaluated their educational environment using an instrument called the Questionnaire of Educational and Learning Capitals (QELC) with items designed to measure each of the ten capitals. Participants responded to each item using a 6-point ordinal-level measurement scale ranging from 1 (*I disagree completely*) to 6 (*I agree completely*). Confirmatory factor analysis (CFA) of the responses showed that the items loaded onto one of each of the 10 factors as expected (see Table 1) and that there was substantial covariance between educational and learning capitals, with typical values of covariance exceeding 0.8.

Since the publication of these seminal research papers, other validation studies have substantiated the factor structure of the QELC with Australian (Lafferty et al. 2020) and German (Mendl et al. 2021) university students, primary-aged gifted Mexican students (Coronel et al. 2021), Israeli primary school students (Paz-Baruch 2015), Turkish gifted and non-gifted students (Leana-Taşcılar 2015a, 2015b, 2016), Iranian upper primary and lower secondary students (Niknam and Baloğlu 2021), and Greek primary and secondary students (Gari et al. 2021).

In extending this research beyond the learner’s actiotope, the Family Educational and Learning Capitals Questionnaire (FELCQ), a variation of the QELC, focused on parental evaluations (*n* = 1917) of their child’s educational and learning capitals, including parental aspirations (Phillipson et al. 2017, 2018). Again, items loaded as expected onto each of the 11 factors with co-variance values between educational and learning capitals exceeding 0.8 (Phillipson et al. 2017). Furthermore, Phillipson et al. (2018) transformed the raw survey responses into Rasch scores (logits) for further analysis and found variability in parental evaluations based on their self-identified cultural background and links between cultural background and the capitals considered important in their children’s educational achievement. For example, Australian parents considered *actional* and *episodic* learning capitals as important in numeracy, whereas Indian parents considered *attentional* learning capital as most important.

In addition to the QELC and the FELCQ, a teacher version has been designed to measure teacher perceptions of their student’s actiotope (Paz-Baruch 2017). Called the Teachers Checklist of Educational and Learning Capital (TCELC), teachers responded to a reduced number of items using three response options, including *not true*, *partly true*, and *totally true*. According to Paz-Baruch (2017), teacher evaluations of the student’s actiotope are dependent on the skill level of the students, where teachers rate students with higher mathematics skills to have significantly more educational and learning capitals than students with lesser skills.

**Figure 1:**
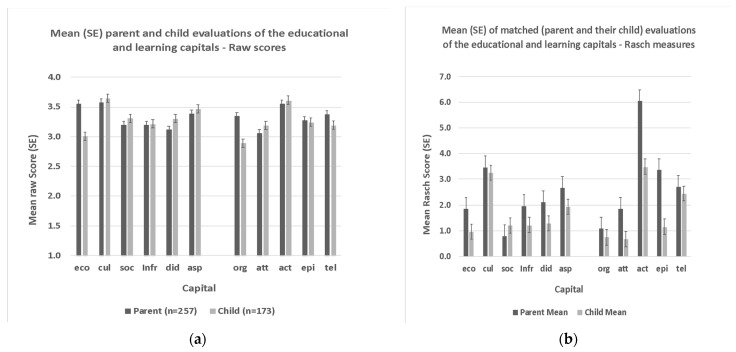
Comparison of mean raw and Rasch scores from parent and child evaluations of their educational and learning environments. (**a**) Saudi parents’ and their gifted children’s evaluations of the gifted educational and learning environment. Values are mean (and SE) of raw scores based on a 4-point scale: 1 = Completely disagree, 2 = Disagree, 3 = Agree, and 4 = Completely Agree. (**b**) Matched pair comparisons (*n* = 98 Saudi parents and their gifted children) of the Saudi gifted educational and learning environment. Values are Rasch scores. Figure 1a,b reproduced with permission from Alamer et al. (2022).

A recent study involving Saudi gifted students and their parents was based on a Saudi-language version of the QELC, including parental and student *aspirations* (Alamer et al. 2022). Not only did this study match student responses with that of their parents, it also directly compared the usefulness of Rasch scores rather than the means of raw scores (Figure 1a,b). When raw scores were used as the basis of statistical analysis, the results showed that Saudi parents evaluated *economic* and *organismic* capitals higher than their gifted children.

In part, Rasch modeling evaluates the capacity of the instrument to measure variability in participant responses by identifying misfitting items and categories. After omitting any misfitting items and/or categories, Rasch modeling produces objective estimates of participant ‘ability’ based on an interval-level measurement scale termed the logit (e.g., Alamer et al. 2022; Bond and Fox 2015). In Alamer et al., the use of Rasch scores enabled an objective comparison of parents’ and their children’s evaluations of the child’s educational and learning environment. With the exception of *social* educational capital, parents evaluated all capitals as higher than their children’s, with effect size estimates ranging from small (*infrastructural*, *didactic*, and *aspirational*), medium (*economic*), and large (*attentional*, *actional*, and *episodic*). Given the interval-level nature of Rasch scores, it is possible to argue, for example, that parents evaluated the availability of *actional* capital (6.1 logits) as almost double that of their children (3.5 logits). This comparison is not possible with raw survey scores because the intervals between ordinal-level data are largely without meaning.

### 3.2. Links between Capitals and Academic Achievement

With increasing confidence in the overall validity of the QELC across different cultural contexts and levels of education, researchers have turned their attention to a better understanding of the link between educational and learning capitals, academic achievement, and the regulatory processes within the system. For example, Paz-Baruch (2017, 2020) used correlational analysis to show that variability in the learning capitals and general intelligence of Israeli students were linked with mathematics achievement. In these studies, Paz-Baruch showed that the teacher and student evaluations of the availability of the capitals varied according to student levels of mathematical achievement. Interestingly, for students with *high* or *regular ability*, their evaluation for some capitals were negatively correlated with their general intelligence scores measured using a shortened version of the Raven’s Advanced Progressive Matrices test. Although these results are interesting, the conclusions should be viewed cautiously because of the untested validity of a 3-point rating scale, the use of raw scores, and the problems associated with implying causality from correlational analysis.

Using regression analysis, Leana-Taşcılar (2015a) showed that for 4th Grade Turkish students, both *infrastructural* capital and extrinsic motivation predicted their academic achievement. On the other hand, *economic* and *didactic* capital and measures of both intrinsic and extrinsic motivation were important predictors for 7th Grade students’ academic achievement. Extending the research to investigate gender-based differences, (Leana-Taşcılar 2015b) found that girls scored higher than boys in *economic*, *cultural*, *social*, *organismic*, and *telic* capitals compared to boys and that the educational achievement of younger students were more impacted by capitals compared to their older companions. Finally, Leana-Taşcılar (2016) found that in general, gifted students scored lower in all capitals compared to their non-gifted counterparts.

In the Australian context, Phillipson et al. (2018) showed that firstly, parental evaluations of their children’s educational and learning capitals were dependent on their self-identified cultural and ethnic group. For example, Chinese, Indian, and ‘Other Asian’ parents viewed their children’s cultural educational capital as significantly higher compared to Australian, British, and ‘Other European’ parents’ perception. Phillipson et al. also found differences across cultural and ethnic groups in their evaluations of their children’s *organismic*, *actional*, *telic*, *attentional*, and *aspirational* capitals.

Secondly, Phillipson et al. (2018) showed that the capitals that predicted their children’s level of numeracy varied according to their cultural and ethnic groups. For example, Australian parents’ perceptions of their children’s *actional* and *episodic* capitals positively predicted their children’s numeracy scores, while *social* capital was a negative predictor. In contrast, Indian parents’ perceptions of their children’s *actional* and *attentional* capitals were positive predictors of their children’s numeracy scores, and *infrastructural* capital was a negative predictor.

Returning to Susie, the research outlined thus far suggests that her teachers should measure the levels of her educational and learning capitals to try and identify those capitals that are in a relatively lesser amount compared to other capitals. In Susie’s context, it is reasonable to suggest that her motivation appears low, and so they expect that her attentional and telic may be low. Furthermore, they want to check on her mental health to see if there are any issues of bullying. Finally, they also measure the educational and learning environments of her peers in order to get a sense of what a normal actiotope looks like for low-performing, average, and high-performing students.

### 3.3. Investigating the Systemic Nature of the AMG

Paz-Baruch (2020) extended her previous research using SEM to include possible mediation effects. Paz-Baruch concluded that for German students, 80% of the variability in students’ academic achievement can be explained by their general intelligence scores and their assessments of the educational and learning environment. A detailed analysis showed that the effects of educational and learning capitals were greater than general intelligence scores and that the impact of educational capital on academic achievement was mediated by learning capitals. In other words, the positive effect and predictability of educational capitals on academic achievement were amplified and facilitated through learning capitals.

In addition to predicting academic achievement, other research has investigated the regulatory mechanisms supporting the development of the actiotope. For example, system stability is dependent on interactions between educational and learning capitals, and the effects of educational capitals on both robustness and resilience (homeostasis) as well as system growth (allostasis) are mediated by learning capitals (Vladut et al. 2016).

More recently, Mendl et al. (2021) used the 10 educational and learning capitals as part of a broader investigation focusing on the impact of feedback on university students’ motivation and emotions. Using a correlational approach, the researchers found that the effects of feedback on motivation and emotions were not strong. However, they found both educational and learning capitals exerted strong direct effects on feedback, where higher capital scores predicted more favorable responses.

Although research into the interaction effects is tentative, it suggests that the regulatory mechanisms within the actiotope do not vary. In other words, the interactions between capitals do not seem to change from one actiotope to another. Returning again to Susie, the constancy in the interactions suggests that the impact of, for example, changes in attentional learning capital on actional and episodic learning capital is common across all children. In practical terms, this means is that it is possible to investigate the interactions within an actiotope using large sample sizes.

## 4. Methodological Considerations in Current AMG Research

In this section, we summarize what we believe are some of the methodological issues in AMG research to date. Some of these issues are peculiar to AMG research, while others are more general in nature. We also outline our recommendations for future research to overcome some of these issues.

Setting aside the ‘mapping’, intervention, and conceptualization research, the research to date relies on variations of an instrument first described in Vladut et al. (2015, 2016). This instrument requires participants to respond to items that reflect their perceptions regarding the presence of each of the 10 (and later 11) capitals using a 6-point (or 4-point) Likert-like scale. Validation studies using both CFA and Rasch modeling show that the instrument is based on a 10-factor (or 11-factor) structure and has adequate reliability and construct validity.

It is important to bear in mind that parametric tests of statistical significance and modeling assume that the data are derived and reported on an interval-level measurement scale. As we have seen, much of the research to date relies on the collection and analysis of data based on an ordinal-level measurement scale (i.e., a 6-point Likert-like scale). Even if mean scores (and SDs) are calculated, the impression of interval-level data is misleading.

More importantly, the nuanced information contained within participant responses to items and categories of the QELC, for example, is not realized if raw scores are used (Alamer et al. 2022). As Bond and Fox (2015) and others have argued, there is a need for objective measurements in the social sciences with Rasch modeling used to ensure that both items and categories capture the full variability in participant responses. Furthermore, Rasch modeling converts ordinal-level data into interval-level data (logits) and hence fulfills some of the assumptions behind the use of parametric statistical tests.

Recommendation 1. Rasch modeling should be used to develop and validate the items and categories that are used to measure variability in capitals and to measure a person’s ‘ability’ on an interval-level measurement scale.

Given the universal (and mostly unquestioned) reliance of perceptions in actiotope research (Alamer et al. 2022), it is pertinent to consider whether or not participant perceptions are adequate measures of the availability of these capitals or whether other measures may be more relevant. The question becomes much more important when the age of the participants is considered, with younger participants possibly unable to adequately evaluate the availability of *cultural*, *economic*, and *social* educational capitals, for example. Although a close reading of Ziegler’s description of the capitals (e.g., Ziegler and Baker 2013) does not provide a definitive answer to this question, the clear impression is that Ziegler intended that objective measures of the availability of the capitals underpins their measurement.

The distinction between ‘perceptions’ of the availability of capitals and ‘objective measures’ of their availability is important, especially when it comes to SEM^2^. In some of the capitals, it is appropriate to consider other sources of information regarding the availability of capitals. For example, it may be more appropriate to consider combinations of parental assessments of their child’s *cultural*, *economic*, and *infrastructural* capitals; teacher evaluations of their student’s *organismic* and *attentional* capitals; and student assessments.

Recommendation 2: Evaluations of a child’s educational and learning environment should be obtained from a number of sources, including parents and teachers. It is also important to ensure that data sets capture the full variability in capitals and in talent.

As Paz-Baruch’s (2020) single study has shown, developmental models of academic achievement that include aspects of general intelligence (fluid *g*) and capitals explain around 80% of the variability in academic achievement. This is a remarkable result that highlights the need to replicate the model in different contexts to assess the external validity of the finding.

Recommendation 3: Developmental models of academic achievement should include aspects of intelligence, particularly estimates of the child’s fluid *g*.

A close reading of the primary sources surrounding the AMG indicates that the research to date fails to fully account for Ziegler’s (Ziegler 2005; Ziegler and Baker 2013; Ziegler et al. 2017; Ziegler and Stoeger 2017) premise that talent develops within an actiotope. In using SEM to operationalize the AMG, researchers seem to have erroneously assumed that talent—as measured by academic achievement—somehow arises from the capitals themselves. In contrast, it is clear from Ziegler’s various descriptions that higher levels of talent arise from previous levels of talent. Accordingly, interaction between capitals and talent should focus on changes in talent *r*ather than measures of talent at one point in time. In other words, the actiotope can be measured at Time 1 and again at Time 2, and changes in the capitals between Times 1 and 2 could be used to account for changes in talent. Conceptually, this shown in the following formula:

$$tal_{2}\propto tal_{1}\int^{\text{​}}\overset{11}{\underset{i=1}{\sum }}\Delta c_{i}$$

where $tal_{2}$ and $tal_{1}$ are the individual’s level of talent at Time 2 and Time 1, respectively, and $\Delta c_{i}\;$ represents the change in each of the 11 capitals over the same period of time. Rearranging this formula shows that tal2tal1 is proportional to the change in capitals, helping to realize Ziegler et al.’s (2013) goal of being able to quantify the likely impact on talent for any change in capital. At this stage, it is important to note that the sum total of the capitals is subject to some function. However, it is also possible that each of the 11 capitals is subject to their own function or that it is a multiplicative total (∏c) rather than a sum total (∑c) or a combination of the two. Finally, it is also possible it is neither.

For reasons already outlined, we view talent as a latent variable that is measured imperfectly by school-based academic achievement. For Susie’s parents and teachers to better understand what is happening, it is important to have measured the change in capitals as well as the change in her academic achievement. Any changes in capitals could identify possible reasons for the decline in mathematics achievement.

At best, however, academic achievement scores rank students against other students within the same year level. In Year 6, for example, Susie was top of her year level but is now ranked near the bottom in Year 8. However, it is necessary to ensure that researchers are cognizant of the limitations of using academic achievement as an indicator of talent, particularly if the research includes cohorts of several year levels. If talent is viewed as developmental, then Susie’s 99% mathematics achievement score in Year 6 cannot be directly compared with her achievement score of 35% in Year 8. What is needed is an absolute measure of talent in mathematics in Year 6 in order to compare her talent across Years 6–8. Again, Rasch modeling allows the researcher to link important skills in tests of mathematical achievement, thereby showing changes in skills and knowledge over time (Bond and Fox 2015).

Recommendation 4: Models of the AMG should focus on changes in talent (Δtal) as the dependent variable rather than talent at one point in time.

Much of the research linking capitals with academic achievement is based on the use of SEM. Many studies investigating the variables that impact academic achievement currently combine the techniques of meta-analysis with SEM^3^. For example, Lin and Powell (2022) concluded that student variability in several factors, including initial *fluency in mathematics*, *reading*, *working memory*, *attention*, and *self-regulation*, were positively linked with *mathematics performance*. Their data showed that *student age* positively moderated the effects of initial *fluency* and *working memory* on subsequent *mathematics performance*, whereas the impact of *attention* and *self-regulation* declined with age.

A recent study illustrating mediation effects suggests that *mental well-being* (*self-esteem*, *self-image*, *self-efficacy*, and *stress)*, and *healthy behaviors* (*bed-time* and *diet*) might be potential mediators in the relationship between physical activity and academic achievement (Visier-Alfonso et al. 2022). In other words, the impact of physical activity on academic achievement is facilitated and enhanced by mental well-being and healthy behaviors.

In a third study, Hsieh (2022) found evidence for the relationship between *positive* involvement of Asian parents and their children’s academic achievement. Furthermore, the child’s *study habits* positively mediated the effects of *parental involvement*. This means that variability in the child’s *study habits* positively affected their parents’ *involvement*, which in turn positively influenced the child’s academic achievement.

As these three recent examples show, the interaction effects (moderation and mediation^4^) involve a number of variables and an a priori (or hypothesized) relationship between these variables. Structural equation modeling then tests the hypothesized relationships by examining the statistical fit between the hypothesized and the empirical (or data-driven) model.

Despite the value of SEM in educational research, there are still limitations associated with its use. Firstly, as both the number of variables increases and the relationships between variables becomes increasingly non-linear, it becomes increasingly difficult to conceive a hypothesized model that captures the true relationship between variables. Even if it were possible to conceive of a complex model with a large number of variables, a very large sample size would be required to construct such model, especially when criteria such as power, bias, and propriety are taken into account (Deng et al. 2018; Wolf et al. 2013). Furthermore, many of the latent variables researched in educational research are non-normally distributed (Bono et al. 2017), and non-normally distributed data increases the number of observations required for robust parameter estimation in SEM models, thereby placing further demand on large sample sizes (Deng et al. 2018).

The second limitation involves the relationships between the variables. Structural equation modeling assumes that the causal relationship between the variables, whether latent or observed, is essentially linear. Although SEM models are able to take into account measurement errors, the assumption of linearity is fundamental to constructing and interpreting the SEM model. What this means, for example, is that nuanced relationships between variables that are logarithmic (e.g., a∝log(b)) are interpreted as linear but with larger measurement errors.

Finally, the SEM models of the AMG erroneously assume that the interactions between the educational and learning capitals are linear. Moreover, the SEM models do not allow the study of the interactions between individual capitals such as *cultural* capital and *organismic* capital or between *didactic* capital and *attentional* capital in the development of talent, for example. Irrespective of the child and their context, however, the nature of these interactions is not captured by SEM and remains unknown. If these non-linear interactions between capital are identified and quantified, then it is possible to predict the potential changes in talent if changes in the capitals were to be introduced.

Recommendation 5: Mathematical models of AMG should enable interactions between the full range of capitals and take into account the possibility of non-linear interactions.

To summarize, we propose that future research should focus on the interactions between capitals and how these interactions lead to changes in talent. Given the full range of recommendations, we propose a research agenda that extends beyond solely relying on SEM and includes the use of artificial neural networks (ANNs). In the next section, we briefly outline the technology before describing a number of approaches that utilize ANNs.

## 5. Clever Solutions—Artificial Neural Networks and the AMG

Initially developed in the 1950s, the methodology behind artificial neural networks are now in their third generation (Feng et al. 2019). In broad terms, ANNs are machine learning algorithms that are based on the neural networks found in human brains and designed to mimic human learning. Today, they form the basis of artificial intelligence where decisions are required in response to changes in the physical environment and to model complex relationships between variables. It has been used to model enzyme activities in cancerous cells (Burden and Winkler 2008), predict stock prices (Sun et al. 2014), and estimate material weakness in engineering (Feng et al. 2019). Furthermore, ANNs have been used to model reflective thinking (Kayri 2016).

Artificial neural networks have a number of advantages over traditional methods such as SEM. Firstly, ANNs can model complex non-linear relationships between independent and dependent variables without the need for a hypothesized model (Burden and Winkler 2008). In fact, the purpose of the ANN is to ‘learn’ what this relationship is (Kayri 2016). Secondly, ANNs are suitable for large data sets, even when the number of independent variables exceeds the sample size (Okut 2016).

In using ANNs to predict defects in stainless steel production, for example, Feng et al. (2019) was able to find and model the relationships between 22 input variables (16 elements plus 6 physical parameters) and 1 output variable (susceptibility to material cracking) contained within a relatively small data set of 487 observations. More importantly, the model was able to accurately predict defects in stainless steel based on variability in the input variables.

Despite being used in a number of domains, ANNs are only starting to emerge in the education field. Yağcı (2022) identified six research studies that used ANNs and five studies that used other machine learning algorithms to predict students’ academic achievement. Of the six studies that used ANNs, four studies predicted the grades and/or degree progression of university students (Asif et al. 2017; Musso et al. 2020; Waheed et al. 2020; Xu et al. 2019). The remaining two studies reviewed in Yağcı (2022) used ANNs to predict the academic performance of secondary school students (Cruz-Jesus et al. 2020; Hoffait and Schyns 2017). In this section, we outline the basic architecture of an ANN and the important parameters that are under the discretion of the researcher. We then propose how ANNs can be used to better understand the AMG, thereby enhancing the predictive potential of the AMG.

### Basic Structure of Artificial Neural Network

**Figure 2:**
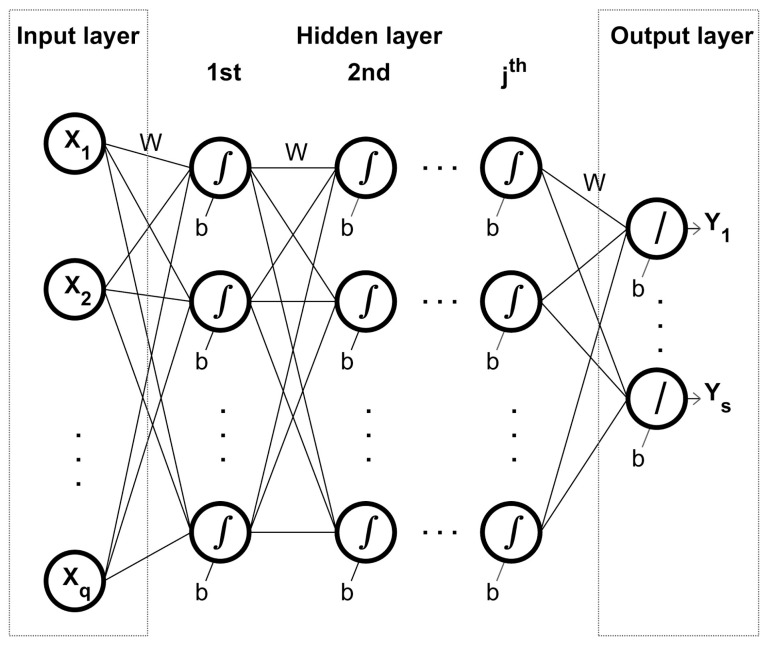
Broad framework of an artificial neural network with j hidden layers. The three broad features of the ANN include input layers receiving information from q input variables. Information flow is from left to right, with activation functions (∫ or/) chosen by the researcher changing the scalar values of information. As the artificial neural network ‘learns’, the values of each connection weight (W) and bias (b) will be updated so that the match between the dependent variables in the data set and the variables in the output layer (Y) are optimized. See text for further details.

The basic architecture of ANNs include neurons arranged in three broad layers (Figure 2). The first layer is termed the input layer; the second layer is termed the hidden layer and in fact may contain more than one layer. The final layer is termed the output layer.

The primary unit of an ANN is the neuron. In Figure 2, each neuron is represented by a circle and is linked to other neurons in the adjacent layer(s), with information flowing from left to right. Information between neurons is weighted (W) and represented by a solid black line, with weightings estimated for each connection. Hence, the input of each neuron in the hidden layer is the sum total of the weighted information from preceding neurons.

As well as receiving information, each neuron in the hidden layer transforms the information using an activation function chosen by the researcher and an associated bias (b) assigned by the model. Under default conditions, the input variables are transferred one set at a time to neurons within the input layer, transformed by the neuron in the hidden layer, and ultimately transferred to the output layer. Here, the output is compared to the dependent variable associated with each data set. Differences between the values in the output layer and the dependent variable provide the basis for adjustments to both the weightings and bias for each neuron. These adjustments are made through learning functions, again chosen by the researcher. In other words, the network learns and estimates the appropriate relationship between the independent and dependent variables.

For a given architecture and data set, the adjusted weightings and biases produce an output value that is then compared with the independent variable using a Pearson correlation (R) (Train-R). Finally, a portion of the complete data set is retained and used to estimate the Test-R. Ideally, the Train-R and Test-R should be comparable in value.

The challenge for researchers is to find the optimal architecture for each data set with a number of network parameters that need to be specified by the researcher. Each of these is outlined in turn.

1. Number of neurons in the input layer—usually, the number of neurons in the input layer is the number of input variables. In the case of the AMG, this means that there would be 10 (or 11) neurons corresponding to the 10 (or 11) capitals. Additional input variables such as IQ score, gender, and year level could be added where deemed necessary. Here, we recommend Rasch-standardized survey scores for each capital and one-hot coding for gender. Although the year level is nominal information, it can be coded as interval-level data.
2. Number of hidden layers—the general guideline is that fewer is better as neural networks with one hidden layer can map most relationships between input and output variables as long as there are sufficient neurons (Hornik et al. 1989). However, in some instances, models with two hidden layers outperform their one-hidden-layer counterparts (Thomas et al. 2016). Therefore, we advise researchers to test models with one and two hidden layers.
3. Number of neurons in each hidden layer—the general guideline is that the number of neurons in each hidden layer should be fewer than the preceding layer (Huang 2003; Stathakis 2009). In practical terms, Thomas et al. (2016) provided guidelines for a ‘short-cut trajectory’ to identify the optical number of neurons within each hidden layer for ANNs with two hidden layers.
4. The number of neurons in the output layer—the number in this layer is simply the number of output variables.
5. Activation function—as information is transferred through each neuron, it undergoes a transformation. This transformation is referred to as an activation (or transfer) function and is selected to fit the values of the input and outputs of the ANN. Commonly used activation functions include linear, hard-limit, and log-sigmoid (Beale et al. 2017). Further choices of activation functions are listed in Table 2.1 of Hagan et al. (2014).
6. Training function—the training function refers to how the weightings and biases are adjusted for each cycle. Using the appropriate parlance, researchers need to decide which back-propagation method is used, with common options including Levenberg-Marquardt and Bayesian regularization (Beale et al. 2017; Okut 2016).
7. Specifying the proportion of the data set as training set and test set—usually, the complete data set is randomly divided into a training set (i.e., 70%) and test set (i.e., 30%). However, this proportion can be adjusted if necessary.

Evaluations and selection of the best ANN architecture is generally reliant on comparing the Train-R with the Test-R. Using the weightings and biases obtained after the training is completed, the independent variables in the test data set are transformed and the output variables compared with the associated dependent variables. A close match between the Train-R and Test-R indicates which ANN architecture best models the relationship between all variables.

In developing an ANN architecture that best models these relationships, researchers need to be alert to three issues: overfit, vanishing gradient, and computational load. Overfit generally occurs when the ANN architecture is overly complex and contains too many hidden layers (and associated neurons). Overfit is evident when the Train-R is much larger than the Test-R.

Vanishing gradient (or inability of the architecture to learn) refers to the failure of the back-propagation algorithm to adjust the weights and biases in the hidden layer closest to the input layer. Again, reducing the complexity of the ANN architecture reduces the issue of vanishing gradient. Finally, computational load refers to the time required to fully train an architecture. The computation load increases exponentially as the ANN contains more neurons and hidden layers because there are more weights and biases to be estimated (Thomas et al. 2016).

In Feng et al. (2019), for example, the optimal architecture was 21-(6-5-4-3)-1 containing 1 input layer with 21 neurons (corresponding to 21 input variables); 4 hidden layers with 6, 5, 4, and 3 hidden neurons, respectively; and 1 output layer consisting of 1 neuron. The researchers selected the hyperbolic tangent function as the activation function for the input and hidden layers, linear for the output layer, and Bayesian regularization as the learning function. Importantly, the values of the Train-R and Test-R were 0.99 and 0.93, respectively. Based on the optimal architecture, Feng et al. (2019) were able to use the associated weights and biases to accurately predict defects in stainless steel structures based on proposed changes in their chemical composition.

## 6. Approaches to and Benefits of Using Artificial Neural Networks to Model the AMG

We propose the following research approaches, beginning with our five recommendations (i.e., *use of Rasch scores*, *increasing number of data sources*, including *measures of intelligence*, measuring *change in talent*, and use of ANN*s*). To emphasize our main argument, we suggest that future research be based on ANNs in order to ‘learn’ and articulate the nature of the interactions between the capitals and talent development between Time 1 and Time 2. Hence, the main objective is to generate data that best match the educational and learning environment and to identify the optimal architecture that best models these interactions. Once identified, we can predict likely changes in talent development if changes in Susie’s environment were to be implemented.

**Figure 3:**
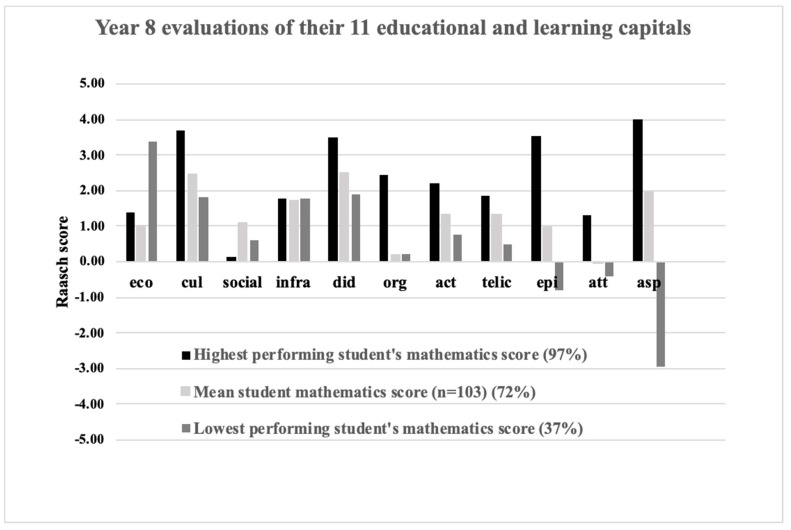
Year 8 student evaluations (*n* = 103) of their 11 educational and learning capitals. Students from one Australian co-educational school were invited to participate in a research that linked their educational and learning capitals with their academic achievement in mathematics (Han et al. 2023). Responses were obtained from students from Years 5–11 (*n* = 590) using a version of the QELC and converted to Rasch scores (logits) The school provided their most recent school achievement scores in mathematics. The highest-performing Year 8 student achieved a mathematics score of 97% compared to the lowest-performing student (37%). The average student achieved a score of 72%.

To better illustrate our proposal, we used data from an unpublished research that identified the highest and least performing Year 8 students in mathematics achievement from one Australian school and their perception of the availability of the educational and learning capitals amongst Australian high school students (Han et al. 2023). Using a variation of the QELC, Han et al. created Rasch scores for each of the 11 capitals for these two students and compared them with the average Rasch capital scores of their classmates (Figure 3).

The differences between the highest- and lowest-performing students lie in several of the capitals, including *organismic*, *telic*, *episodic*, *attentional*, and *aspirational* capitals. In particular, a teacher with such information might conclude that for the lowest-performing student, the availability of *economic* capitals is not problematic, but the teacher should support the student and their family in focusing on their educational *aspirations*, the student’s *attention* in class, their goals (*telic* capital), and their physical and mental health (*organismic* capital), and help the student apply their knowledge in new contexts (*episodic* capital).

Returning now to Susie: in order to understand the reasons for the decline in her academic achievement, we are interested in knowing which capitals seem to be problematic. After profiling her capitals, we see that her *aspirations*, *attention*, and *goals* are considerably lower than other students. Her teachers want to know if focusing on these capitals is likely to enhance her academic achievement and if so, to what extent^5^. Equally important, her teachers are interested in knowing the likelihood that her achievement scores will increase from current levels (50%) to, say, 75%.

Answers to such questions are based on finding the optimal ANN architecture that best describes the relationship between capitals (and any other demographic variables) and academic achievement. During this process, the current data set is used to estimate the values for each connection weight and bias within this ANN architecture. Moreover, the values of each weight and bias can be retrieved by the researcher and used to estimate the effect of potential changes in capitals on academic achievement.

In Susie’s case, for example, connection weights and biases for the optimal model could be used to estimate the likely effect on her academic achievement if her *aspirations* alone were to be increased. If the prediction failed to show any significant change in academic achievement, the researcher could then investigate the effect of increasing both *aspirations* and *attention* on her academic achievement, and so on. Our preliminary investigations showed that increasing the *aspirations* of the least able student to a level approaching the most able student also increased their predicted academic achievement.

Given that previous research linking capitals and academic achievement have relied on SEM, we suggest that initial efforts using ANNs should be based on current research designs. In other words, participants^6^ should be asked to evaluate the availability of the capitals at one point in time (Time 1) and their responses modeled against academic achievement using both SEM and ANNs. Of course, an important aspect of this work will be to identify the optimal ANN architecture with the highest predictability for academic achievement.

Directly using both techniques to analyze the same data will assist in the interpretation of ANNs. In particular, we note the potential of SEM to help explain the variability in academic achievement, and we expect that the optimal ANN architecture will have several features that are congruent with the SEM model. These features include the identification of the most important capital(s) in the prediction of academic achievement. However, we expect that the explanatory power of the optimal ANN architecture will exceed its corresponding SEM model.

For reasons already outlined, we recommend that research designs that are based on the use of ANNs and two distinct time periods will better reflect the theoretical basis of the AMG. Accordingly, we suggest assessing academic performance at Time 1 and at Time 2 and evaluating the child’s educational and learning capitals at Time 2 from a variety of sources, including from their parents and teachers. We recommend the independent (input) variables for analysis to include their educational and learning capitals at Time 2 and the child’s academic achievement scores at Time 1, with the re-assessed academic performance achievement at Time 2 as the dependent variable. In other words, the ANN that models and predicts academic achievement at Time 2 would depend on both the child’s baseline academic achievement and their current education environment.

Such modeling will produce weights and biases that reflect the complex interactions between capitals as they act on previous levels of talent. We assume that the underlying interactions between capitals and academic achievement will be common across all participants and that different capital scores reflect differences in the availability of the capitals and not the nature of their interactions with academic achievement. Accordingly, it would now be possible to predict what could have happened in the time frame between Time 1 and 2 if there were changes in one or more of these capitals. Using the data for the low-performing student (Figure 3), for example, the likely impact of enhancing the child’s aspirations and episodic capitals on their academic achievement could be estimated. If the optimal ANN model shows that changes in *aspirations* and *episodic* capitals doubled their predicted mathematics score, the student’s teacher and school will have a strong evidence-based foundation for intervention.

More broadly, school administrators could use the optimal ANN models to inform schoolwide interventions that are designed to enhance the overall performance of its students. Using the example from Figure 2, such interventions could include partnerships with parents that focus on the child’s physical and mental health (*organismic* capital), their immediate learning goals (*telic* capital), and their aspirations for higher education (*aspirational* capital). We emphasize that these interventions should be evidence-based when they are linked to improving academic performance. Furthermore, school administrators should be aware that small changes in one or more of the other capitals may further promote the desired outcomes.

## 7. Concluding Remarks

In conclusion, we consider the AMG as the most useful model to date to explain the development of skills and knowledge that ultimately may lead to exceptionality. In agreement with many other developmental models, the AMG includes a combination of cognitive and non-cognitive factors. In contrast to other models, the AMG takes a systems perspective in which exceptionality is the end-point of a (usually) long developmental journey. For students such as Susie, the likelihood that she will achieve her dreams of becoming an engineer depends on the continual adaptation of an actiotope that supports the skills and knowledge she needs to complete her education. Whether Susie achieves exceptionality requires the further adaptation of her actiotope.

In many ways, the question of Susie’s exceptionality is largely irrelevant for her parents and teachers. What is important for Susie, her parents, and teachers at the early stages of her development is to identify the aspects of the educational and learning environments that prevent the continual adaptation of her actiotope. Once identified, the objective is to change the environment in order to optimize her actiotope for learning.

Because an actiotope is complex, the challenge for researchers to understand the interactions between the educational and learning environments and talent development is immense. Although some progress has been made, we believe that current approaches to research have not yet fully reflected this complexity. Despite the pessimism expressed in Ziegler et al. (2017), we believe that the AMG is amenable to empirical testing and that ANNs are the ideal technology to do so. In particular, we suggest that ANNs can be used to model the non-linear interactions between the capitals and academic achievement. We propose that future research using ANNs can provide teachers and school administrators with evidence-based interventions that can enhance academic performance for students such as Susie and support administrators with the tools to be more efficient in the reallocation of existing capitals in order to enhance overall school performance.

## Figures and Tables

**Table 1:** The five exogenous and five endogenous capitals plus aspirations in the actiotope model ^1^.

Exogenous Capitals	Endogenous Capitals
Economic educational capital (eco) includes every kind of wealth, possession, money, or valuable that can be invested in the initiation and maintenance of educational and learning processes.	Organismic learning capital (org) consists of the physiological and constitutional resources of a person.
Cultural educational capital (cul) includes value systems, thinking patterns, models, and the like that can facilitate or hinder the attainment of learning goals.	Actional learning capital (act) denotes the action repertoire of a person—the totality of actions they are capable of performing.
Social educational capital (soc) includes all persons and social institutions that can directly or indirectly contribute to the success of learning.	Telic learning capital (tel) comprises the totality of a person’s anticipated goal states that offer possibilities for satisfying a person’s performance.
Infrastructural educational capital (infra) relates to materially implemented possibilities for action that permit learning to take place.	Episodic learning capital (epi) concerns the simultaneous goal- and situation-relevant action patterns that are accessible to a person.
Didactic educational capital (did) means the assembled know-how involved in the design and improvement of learning processes.	Attentional learning capital (att) denotes the quantitative and qualitative attentional resources that a person can apply to learning.
Aspirational educational capital (asp) refers to the value placed on higher education.	

^1^ The definitions were adapted from Ziegler and Baker (2013), Phillipson et al. (2017), and Phillipson et al. (2018).

## Data Availability

No data was generated from this research.

## Notes

1 Ziegler and Baker (2013, p. 35) preferred the term *capital* over *resource* becaue capitals could take negative values, are interchangeable with other capitals, generally need to be ‘earned’ and can grow.

2 Irrespective of this distinction, SEM assumes that measures are independent of each other (Byrne 2016; Morrison et al. 2017). In other words, data sets that are based on individual evaluations of all capitals contravene the assumption of data independence and, strictly speaking, should not be used in SEM.

3 There are two types of SEM, including covariance-based (CB) SEM and partial least squares structural equation modeling (PLS-SEM) (cf. Hair et al. 2021). Being the first to be developed, CB-SEM techniques seem to be more commonly used in education-related contexts whereas PLS-SEM is used more in business-related contexts. The strengths and limitations of each type of SEM are outlined in Hair et al. (2021).

4 Moderating variables influence the action of an independent variable, whereas mediating variables are intermediate in the link between independent and dependent variables. See Byrne (2016) for a full explanation regarding the differences between moderation and mediation.

5 Indeed, Ziegler and colleagues have often posed similar questions. For example, Ziegler et al. (2018) asked *What are the chances that students from Beijing No. 8 Middle School will win at least 20 gold medals at the International Mathematics Olympiad between 2020 and 2030?*

6 We use the term to refer to parents, teachers and/or students.
